# Multimorbidity and its associated risk factors among adults in northern Sudan: a community-based cross-sectional study

**DOI:** 10.1186/s41043-024-00513-7

**Published:** 2024-01-27

**Authors:** Imad R. Musa, Ahmed Ali Hassan, Ishag Adam

**Affiliations:** 1 Royal Commission Hospital at AL Jubail Industrial City, Al Jubail, Kingdom of Saudi Arabia; 2 Faculty of Medicine, University of Khartoum, Khartoum, Sudan; 3 Department of Obstetrics and Gynecology, Unaizah College of Medicine and Medical Sciences, Qassim University, 51911 Unaizah, Kingdom of Saudi Arabia

**Keywords:** Multimorbidity, Hypertension, Diabetes mellitus, Obesity, Anaemia, Depression

## Abstract

**Background:**

Multimorbidity (having two or more coexisting long-term conditions) is a growing global challenge. However, data on multimorbidity among adults in Africa, including Sudan, are scarce. Thus, this study aimed to investigate the prevalence of multimorbidity and its associated risk factors among adults in Sudan.

**Methods:**

A community-based cross-sectional study was conducted in northern Sudan from March 2022 to May 2022. Participants’ sociodemographic characteristics were assessed using a questionnaire. Multimorbidity was defined as having two or more coexisting long-term conditions, including diabetes mellitus (DM), hypertension, obesity, anaemia and depression-anxiety. Multivariate logistic regression analyses were performed to determine the associated factors.

**Results:**

The participants included 250 adults: 119 (47.6%) males and 131(52.4%) females. The median interquartile range (IQR) of the enrolled adults of the age was 43.0 (30.0‒55.0) years. Of the 250 adults, 82(32.8%), 17(6.8%), 84(33.6%), and 67(26.8%) were normal weight, underweight, overweight, and obese, respectively; 148(59.2%), 72(28.8%), 63(25.2%), 67(26.8%), and 98(39.2%) had hypertension, DM, anaemia, obesity, and depression-anxiety, respectively. A total of 154 adults (61.6%) had multimorbidity: 97(38.8%), 49(19.6%), and 8(3.2%) had two, three, and four morbidities, respectively. The remaining 21 (8.4%), and 75 (30.0%) adults had no morbidity, and one morbidity, respectively.

In amultivariate logistic regression analysis, increasing age (adjusted odd ratio [AOR] = 1.03, 95% CI = 1.01‒1.05), and female sex (AOR = 2.17, 95% CI = 1.16‒4.06) were associated with multimorbidity.

**Conclusions:**

The high prevalence of multimorbidity revealed in this study uncovers a major public health problem among Sudanese adults. Our results show that increasing age and female sex are associated with multimorbidity. Additional extensive studies are necessary to evaluate the magnitude of multimorbidity for improved future planning and establishing effective health systems.

## Background

Multimorbidity is defined as having two or more coexisting long-term conditions and it has been reported to have a remarkable increase worldwide in the past two decades [1]. Two main approaches are adopted to address the presence of coexistent medical conditions: comorbidity and multimorbidity. First, the concept of comorbidity implies a disease-specific approach addressing mainly the index condition and leaving other coexisting conditions as less important. Second, the concept of multimorbidity refers to patient-centred approach where all conditions are equally important [2]. Hypertension and diabetes mellitus (DM) representthe common disease clusters and main drivers of multimorbidity worldwide [3]. Several studies across the globe [4] and in countries with fewer resources [5], Middle East countries [6] and Sub-Saharan countries [5, 7] reported different types of prevalence of multimorbidity. Moreover, the prevalence and patterns of multimorbidity are affected by many factors, such as age, gender, obesity and residential regions [3, 8, 9]. Multimorbidity is associated with a huge burden: feelings of dependency, social rejection, psychological distress and poor medication adherence, quality of care and social and sexual health of patients with financial hardships to access optimal medical services for multimorbidity care. In addition, health systems are not appropriately prepared to provide integrated and coordinated care for people living with multiple chronic medical conditions [7, 10]. Hence, assessing multimorbidity and improvingthe understanding of this health problemis crucial for developing and improving the health of communities that involves the patients’ perspective as well as medical education and health system reforms to adopt an integrated health system that promotes partnerships between doctors, community health providers and patients with their families [11]. Although scientific research in Sudan does not focus on multimorbidity, many clinical studies evaluated separately the elements of multimorbidity, e.g. hypertension (40.8%) [12], DM, (18.7%‒30.8%) [13, 14], anaemia (36.2%) [15], obesity (24.5%‒32.7%) [12, 16, 17] and depression/anxiety in patients with DM (35.6%) [18]. Furthermore, hospital admissions and mortality rates related to non-communicable diseases were high among Sudanese people compared with those in other African countries [19]. Although households in Sudan spend the lowest costs for non-communicable diseasecare compared with those in developed countries, these amounts are considerably high for many citizens in Sudan and developing countries [20]. However, little is known about the magnitude and effect of multimorbidity on health-related burdens in Sudan. Moreover, because no recent published data addressing this issue in Sudan are available. Thus, this study aimed to evaluate the prevalence and associated risk factors of multimorbidity among adult Sudanese.

## Methods

### Study area

River Nile State is one of the 18 states in Sudan and recorded a total population of 1,120,441 in the 2008 census [21]. The state has seven localities, described as the lowest administrative unit in Sudan.

## Study population and design

This community-based cross-sectional study was conducted from March 2022 to May 2022 in three villages in the Wad Hamid district located in Almatamah Locality, River Nile State, northern Sudan. The Wad Hamid district borders Khartoum State and is approximately 100 km from Khartoum, the capital of Sudan. The details of the methods are mentioned in our previous work [22].

Almatamah Locality was randomly selected from the seven localities, and among its three districts, the Wad Hamid district was also selected randomly. Three villages were chosen from the randomly selected district using systematic sampling. Then, 80–100 households from each village were recruited based on the population density to obtain the desired sample size. The first member in each household who agreed to participate and met the study inclusion criteria was selected. If the selected house was uninhabited or its inhabitants refused to participate, the next house was selected to meet the target number for the study. In case of two household members were eligible to participate in the study, both of them were selected. The investigators trained three medical officers for data collection to standardise the data collection procedure and maintain data quality. After signing an informed consent form, all adults (male and female) from the households aged ≥ 18 years were enrolled using a lottery method. Participants aged < 18 years, pregnant women, patients with poor cognitive functions and severely ill patients were excluded from this study. As above mentioned, multimorbidity is defined as the coexistence of two or more chronic medical conditions [1, 23].

## Data collection

The present study strictly followed the Strengthening the Reporting of Observational Studies in Epidemiology guidelines [24]. A pretested questionnaire was used during face-to-face interviewsto gather data on sociodemographic characteristics, including age in years, sex (male or female), education (< secondary or ≥ secondary), occupation (employed or unemployed), smoking (never or former/current) and alcohol consumption (never or former/current). In this study, five diseases were identified and included, namely hypertension, DM, obesity, anaemia and depression/anxiety. The identified diseases were diagnosed through self-reporting by the participants and/or newly diagnosed in the current study.

## Blood pressure measurement

Blood pressure was measured using a standard mercury sphygmomanometer with the appropriate cuff size after resting for at least 10 min in a sitting position, with the arm maintained at heart level. The mean of two blood pressure readings (with an interval of 1–2 min) was calculated. If the difference between the two readings was > 5 mmHg, then the measurements were retaken until the reading became stable. Any participant known as hypertensive and receiving treatment or newly diagnosed with hypertension (having a systolic blood pressure of ≥ 140 mmHg or a diastolic blood pressure of ≥ 90 mmHg or both on repeated examinations) was considered as hypertensive [25].

## Anthropometric measurements

Each participant’s weight was measured in kilograms (kg) using well-calibrated scales and was adjusted to zero before each measurement as the standard procedure. The participants stood with minimal movement, hands by their sides and shoes and excess clothing removed. Then, their height was measured in centimetre (cm) after standing straight with their back against a wall and feet together. Body mass index (BMI) was computed as the weight in kg divided by the square of the height in meters (m) (kg/m^2^) [26]. BMI was grouped according to the World Health Organization (WHO) classification into underweight (< 18.5 kg/m^2^), normal weight (18.5–24.9 kg/m^2^), overweight (25.0–29.9 kg/m^2^) or obese (≥ 30.0 kg/m^2^) [26].

## Blood analysis

Blood samples of 5 millilitres collected under aseptic conditions from the participants were transferred to our laboratory for measurement of haemoglobin and glycated haemoglobin (HbA1c) levels. A complete blood count was performed following the manufacturer’s instructions (Sysmex KX-21, Japan). An automated haematology analyser was used to measure haemoglobin levels as described in our previous work [27]. Anaemia was diagnosed following the WHO’s definition of the disease, i.e. haemoglobin concentration of < 12 g/dl in non-pregnant women and < 13 g/dl in men [28]. As described in our previous work, HbA1c levels were measured using an Ichroma machine following the manufacturer’s instructions (Republic of Korea) [12].

## Diabetes mellitus

Patients identified with a diagnosis of DM were those who had documentation of DM (type 1 and 2), whether they were on diet control or glucose-lowering drugs during the time of the study or those who had HbA1c ≥ 6.5% as recommended by the International Diabetes Federation guideline for non-pregnant adults [29].

## Depression-anxiety assessment

In the present study, depression and anxiety were assessed via the Hospital Anxiety and Depression Scale (HADS). HADs is a self-assessment scale developed to assess the presence of depression-anxiety in clinical settings [30]. Furthermore, HADS was tested to be reliable in the general population and has been validated among undergraduates, including medical students. This scale contains 14 items, with 7 items each for the depression and anxiety subscales. Scores for each item range from 0 to 3. Participant scores of 0–7, 8–10 and ≥ 11 indicate a normal condition, borderline depression-anxietyand a probable case of depression-anxiety, respectively. Additional details are described in previous studies [30, 31].

## Sample size calculation

OpenEpi Menu was used to compute the desired sample size, resulting in 250 adults. This sample size of adults was estimated assuminga prevalence of multimorbidity of 20.7% among adults as previously reported in the 2016 demographic and health survey of South Africa [32]. This sample size was calculated to detect a difference of 5% at *α* = 0.05, with a power of 80%.

## Statistical analysis

Data were entered into a computer using IBM Statistical Package for the Social Sciences® (SPSS®) for Windows, version 22.0 (SPSS Inc., New York, United States). The proportions were expressed as frequencies (%). The Shapiro–Wilk test for determining the normality of continuous data (age and BMI) revealed a non-normal distribution. The non-normally distributed data were expressed as the median (interquartile range [IQR]). A univariate analysis was performed for multimorbidity as the dependent variable, (i.e., the dependent variable is categorical) and sociodemographic demographic (age, sex, education, occupation, smoking and alcohol consumption) as independent variables. A multivariate logistic regression analysis was also performed, including all variables with a *p* < 0.2 to control for confounding variables. Sensitivity analyses were performed by running models with each factor of interest separately and we did not observe any difference in results. Adjusted odds ratios (AORs) and 95% confidence intervals (CIs) were calculated as they were applied. A two-sided *p* < 0.05 was considered statistically significant.

## Results

This study recruited 250 participants: 119(47.6%) males and 131(52.4%) females. The median (IQR) of the enrolled adults by age and BMI was 43.0(30.0‒55.0) years and 26.6(23.1‒30.2) kg/m^2^, respectively. Of the 250 adults, 156(62.4%) and 94(37.6%) had ≥ secondary and < secondary education, respectively; 113 (45.2%) were employed and 137 (54.8%) were unemployed; 82(32.8%), 17(6.8%), 84(33.6%) and 67(26.8%) were normal weight, underweight, overweight and obese, respectively (Table 1); and 148(59.2%), 72(28.8%), 63(25.2%), 67(26.8%) and 98(39.2%) were diagnosed with hypertension, DM, anaemia, obesity and depression-anxiety, respectively. A total of 154 adults (61.6%) had multimorbidity: 97(38.8%), 49(19.6%) and 8(3.2%) had two, three and four morbidities, respectively. The remaining 21 (8.4%) and 75 (30.0%) had no morbidity and one morbidity, respectively.

**Table 1:** Comparison of the sociodemographic factors between adults with and without multimorbidity in northern Sudan, 2022(n = 250)

Variables		Total (number = 250)	Adults with no multimorbidity (number = 96)	Adults with multimorbidity (number = 154)
*Median (interquartile range)*				
Age, years		43.0 (30.0‒55.0)	38(26.5‒49.0)	46.5(34.0‒60.0)
*Frequency (proportion)*				
Sex	Male	119(47.6)	56(58.3)	63(40.9)
	Female	131(52.4)	40(41.7)	91(59.1)
Marital status	Married	71(28.4)	33(34.4)	38(24.7)
	Unmarried	179(71.6)	63(65.6)	116(75.3)
Education level	≥ secondary	156(62.4)	60(62.5)	96(62.3)
	< secondary	94(37.6)	36(37.5)	58(37.7)
Occupation status	Employed	113(45.2)	48(50.0)	65(42.2)
	Unemployed	137(54.8)	48(50.0)	89 (57.8)
Smoking	Never	204(81.6)	72(75.0)	132(85.7)
	Current/former	46(18.4)	24(25.0)	22(14.3)
Alcohol consumption	Never	237(96.0)	90(93.8)	147(97.4)
	Current/former	10(4.0)	6(6.2)	4(2.6)

No significant difference was noted in marital status, education, occupation, smoking and alcohol consumption between adults with and without multimorbidity. The median (IQR) of age was significantly higher in adults with multimorbidity than those without multimorbidity (46.5[34.5‒60.0] years versus 43.0[30.0‒55.0]) years, *p* = 0.001]. Compared with the males, the number of females with multimorbidity was significantly higher (63 [40.9%] versus 91 [59.1%], *p* = 0.008) (Table 1).

In the univariate logistic regression analysis, increasing age, female sex and smoking were positively associated with multimorbidity, whereas marital status, education, occupation and alcohol consumption were not associated with multimorbidity. In a multivariate logistic regression analysis, increasing age (AOR = 1.03, 95% CI = 1.01‒1.05), and female sex (AOR = 2.17, 95% CI = 1.16‒4.06) were associated with multimorbidity (Table 2).

**Table 2:** Univariate and multivariate logistic regressionanalysis for factors associated with multimorbidity among adults in northern Sudan, 2022

Variables		Univariate analysis		Multivariate analysis	
		Odds ratio (95% confidence interval)	*P value*	Odds ratio (95% confidence interval)	*P* value
Age, years		1.03(1.01‒1.05)	0.001	1.03(1.01‒1.05)	0.001
Sex	Male	Reference			
	Female	2.02(1.21‒3.39)	0.008	2.17(1.16‒4.06)	0.016
Marital status	Married	Reference			
	Unmarried	0.63(0.36‒1.09)	0.098	1.20(0.64‒2.26)	0.569
Education level	≥ secondary	Reference			
	< secondary	1.01(0.60‒1.70)	0.979	–	–
Occupation status	Employed	Reference			
	Unemployed	1.37(0.82‒2.29)	0.229	–	–
Smoking	Never	Reference			
	Current/former	2.0(1.05‒3.82)	0.033	0.85(0.37‒1.94)	0.692
Alcohol consumption	Never	Reference			
	Current/former	0.41(0.11‒1.49)	0.162	0.58(0.14‒2.40)	0.448

## Discussion

Our study demonstratesa high prevalence (61.6%) of multimorbidity among Sudanese adults; the main factors associated with multimorbidity were increasing age, and being female. The present prevalence (61.6%) of multimorbidity was considerably higher than that reported in certain African countries: Ethiopia (54.8%) [9], Botswana (5.4%) [5], and South Africa (3%–23%) [3]. Similarly, the finding was markedly higher than the overall global prevalence of multimorbidity (37.2%), with the highest prevalence documented in South America (45.7%), North America (43.1%), Europe (39.2%), Asia (35%) [4] and the Middle East (21.8%) [6]. Marked variations in the prevalence of multimorbidity were reported in low- and middle-income countries (LMICs) (3.2%–90.5%)[33]. In addition, the prevalence of multimorbidity was low to moderate (3%–23%) in younger people compared with older adults (30%–87%) [3].

The higher prevalence of multimorbidity identified in our study may be explained by the global growth of multimorbidity over the last two decades [1, 4, 23]. Moreover, the difference in prevalence obtained in various studies is due to the variations in the definitions of multimorbidity, the number of chronic conditions used in different articles, (i.e., certain studies adopted at least two, whereas others considered at least three chronic conditions) [34] and heterogeneity in methodologies [35]. Additionally, the variation in the prevalence of multimorbidity in LMICs could be due to limited epidemiological studies of multimorbidity and even the available ones are conducted in a few countries [33]. Furthermore, the low prevalence in African countries may reflect an underestimation of multimorbidity as many undiagnosed chronically ill patients in Africa are not reported [4].

Following many other studies across the globe, the current study demonstrates a significant association between increasing age and multimorbidity: Ethiopia [36], South Africa [3], Botswana [5], Kenya [37], Burkina Faso [38], Ghana [39], Malaysia[40], China [8] and the Middle East region [6]. A similar significant association between age and multimorbidity was reported in systematic review and meta-analysis studies [4, 10]. Population ageing is driving the worldwide epidemic of chronic diseases, such as cardiovascular diseases, malignant neoplasms, chronic respiratory diseases, and musculoskeletal disorders [10]. This information is based on the outcome of recently published data revealing that multimorbidity was significantly prevalent in the old age group compared with the younger ones [3, 5]. The significant association of age and multimorbidity may reflect an improvement in survival of this age group and earlier and improved detection of diseases in addition to changing lifestyle factors [41]. Moreover, the loss of physical and functional health, including frailty, was associated with ageing and contributed to the multimorbidity status [42]. Furthermore, a component of ageing represents the imbalance between inflammatory and anti-inflammatory networks in individuals, leading to a low-grade chronic inflammatory state known as ‘inflammageing’ [43]. Hence, this vulnerable group of patients face many difficulties, including misplaced global health priorities, ageism, the poor and unprepared health systems to deliver age-appropriate care for patients and challenges in coping with the difficulty of integrating care for complex multimorbidity [10].

Our study shows that female gender is significantly associated with a high risk of multimorbidity. This finding is similar to studies in Kenya [37], Botswana[5, 44], Ghana [39], Burkina Faso [38], the Middle East region [6], India [45], Malaysia [40], and China [8] and with systematic review and meta-analysis studies [4]. The gender difference can be explained by the females’ attitudes and behavioural patterns as women may pay more attention to their health status [46] and are more likely to report their diseases compared with men [47]. Other studies have proposed a greater prevalence of multimorbidity among females, a longer life expectancy and worse health status compared with males [48, 49]. Moreover, hormonal changes in menopausal and postmenopausal periods expose women to an increased risk of many chronic diseases, which may also contribute to a higher prevalence of multimorbidity [50].

Similar to other studies, we demonstrate a non-significant association between multimorbidity and marital status, occupation, education, smoking and alcohol consumption [5, 8, 51, 52]. This finding may point to the heterogeneous process and multifactorial aetiology of multimorbidity that involves ageing, the genetic background, environmental exposures and the inflammatory response of the human body [43]. Multimorbidity exacerbates the huge burden on patients and their families [53], the poor preparedness of health systems to cope with their multimorbidity and the complexity of integrated care for this age group [10]. Hence, these studies including the current study offer essential data regarding the magnitude of multimorbidity and the current challenges to pave the road for planning and establishing integrated health systems. In addition, such studies will contribute to providing person-centred care, prioritising the most concerning the individuals and their carers and ensuring effective, coordinated, minimally disruptive care that is aligned with the patient’s values and safety.

This study has limitations: the nature of study, since it is a cross-sectional study selection biases may exit; it is from one community area and no information was available from the patients admitted to hospitals during the study period; the definition of multimorbidity is not unified due to variations; and certain risk factors were not assessed, such as diet and physical exercise. Moreover, detailed information regarding disease severity, duration of each morbidity, and income was not included due to limited data availability. The data obtained showed a comprehensive multimorbidity pattern, which may increase the prevalence of multimorbidity with increasing age. Finally, certain important diseases that are more prevalent among older people, such as prostate diseases, were not included. This limitation may lead to an underestimation of the multimorbidity prevalence and misclassifications of multimorbidity patterns.

## Conclusions

The high prevalence of multimorbidity revealed in this study uncovers a major public health problem among Sudanese adults. Our results show that increasing age and female sex are associated with multimorbidity. Additional extensive studies are necessary to evaluate the magnitude of multimorbidity for improved future planning and establishing effective health systems.

## Data Availability

Data generated or analyzed during this study are included in this article and are available from the corresponding author on reasonable request.
